# Common carotid segmentation in ^18^F‐sodium fluoride PET/CT scans: Head‐to‐head comparison of artificial intelligence‐based and manual method

**DOI:** 10.1111/cpf.12793

**Published:** 2022-11-16

**Authors:** Reza Piri, Yaran Hamakan, Ask Vang, Lars Edenbrandt, Måns Larsson, Olof Enqvist, Oke Gerke, Poul Flemming Høilund‐Carlsen

**Affiliations:** ^1^ Department of Nuclear Medicine Odense University Hospital Odense Denmark; ^2^ Department of Clinical Research University of Southern Denmark Odense Denmark; ^3^ Department of Molecular and Clinical Medicine, Institute of Medicine, Sahlgrenska Academy University of Gothenburg Gothenburg Sweden; ^4^ Department of Clinical Physiology Region Västra Götaland, Sahlgrenska University Hospital Gothenburg Sweden; ^5^ Eigenvision AB Malmö Sweden; ^6^ Department of Electrical Engineering Chalmers University of Technology Gothenburg Sweden

**Keywords:** artificial intelligence, atherosclerosis, carotids, positron emission tomography

## Abstract

**Background:**

Carotid atherosclerosis is a major cause of stroke, traditionally diagnosed late. Positron emission tomography/computed tomography (PET/CT) with ^18^F‐sodium fluoride (NaF) detects arterial wall micro‐calcification long before macro‐calcification becomes detectable by ultrasound, CT or magnetic resonance imaging. However, manual PET/CT processing is time‐consuming and requires experience. We compared a convolutional neural network (CNN) approach with manual segmentation of the common carotids.

**Methods:**

Segmentation in NaF‐PET/CT scans of 29 healthy volunteers and 20 angina pectoris patients were compared for segmented volume (Vol) and mean, maximal, and total standardized uptake values (SUVmean, SUVmax, and SUVtotal). SUVmean was the average of SUVmeans within the VOI, SUVmax the highest SUV in all voxels in the VOI, and SUVtotal the SUVmean multiplied by the Vol of the VOI. Intra and Interobserver variability with manual segmentation was examined in 25 randomly selected scans.

**Results:**

Bias for Vol, SUVmean, SUVmax, and SUVtotal were 1.33 ± 2.06, −0.01 ± 0.05, 0.09 ± 0.48, and 1.18 ± 1.99 in the left and 1.89 ± 1.5, −0.07 ± 0.12, 0.05 ± 0.47, and 1.61 ± 1.47, respectively, in the right common carotid artery. Manual segmentation lasted typically 20 min versus 1 min with the CNN‐based approach. Mean Vol deviation at repeat manual segmentation was 14% and 27% in left and right common carotids.

**Conclusions:**

CNN‐based segmentation was much faster and provided SUVmean values virtually identical to manually obtained ones, suggesting CNN‐based analysis as a promising substitute of slow and cumbersome manual processing.

## 1 INTRODUCTION

Atherosclerosis is the origin of major cardiovascular and cerebrovascular diseases, (Lorenz et al., 2006; World Health Organization, 2019) which are the number one cause of mortality worldwide despite advancements in diagnostic and therapeutic measures (Barquera et al., 2015; World Health Organization, 2019). Atherosclerotic changes develop in childhood, but rarely cause symptoms until adulthood, in men from age 40−45, in women with a 10‐year delay (Enos et al., 1955; Holman et al., 1958; McGill, 1968). Carotid artery disease is a major cause of stroke, accounting for about 20% of all cases. Carotid artery disease can cause a stroke or transient ischemic attack (TIA) in three major ways: (a) a plaque narrows and completely blocks a carotid artery (total occlusion); (b) plaque rupture damages the lining of the artery with clot formation and finally thrombosis; (c) an emboli on the plaque breaks off and passes with the blood to the brain, where it blocks a brain blood vessel. All three cause an interruption in the blood flow to the brain and can result in symptoms of stroke or TIA (Chambless et al., 2000; Derlin et al., 2011; Wu et al., 2017). Carotid artery disease may be present without symptoms and is usually diagnosed in connection to a stroke or transient ischaemic attack, the identical symptoms of which include weakness in face or arms and speech difficulties. In patients with carotid artery disease, atherosclerosis may develop also in other arteries throughout the body (Lorenz et al., 2006). After the emergence of symptoms, atherosclerotic changes may be detected in plaque form with or without calcification, primarily using structural imaging modalities, such as conventional X‐rays, ultrasound, CT and magnetic resonance imaging (Høilund‐Carlsen et al., 2021). The diagnostic modalities are seldom utilized routinely in individuals with asymptomatic atherosclerosis and often fail to detect atherosclerotic plaque unless tissue change is relatively macroscopic (Prabhakaran et al., 2007).

Positron emission tomography (PET) allows detection of atherosclerosis by tracking microscopic tissue changes, before conventional imaging modalities can detect them (McKenney‐Drake et al., 2018; Raynor et al., 2016). For example, ^18^F‐sodium fluoride (NaF) maps microcalcification (Derlin et al., 2011; Høilund‐Carlsen et al., 2020; Sorci et al., 2020) and thus, NaF detects microcalcification, a crucial feature of atherosclerosis. However, PET imaging also has limitations. Analysing PET scans is a relatively time‐consuming process depending on the target organ. However, artificial intelligence (AI) models in the shape of image analysis models, may overcome this limitation as observed in other diseases including cancer (Lindgren Belal et al., 2019; Dou et al., 2017). More specifically, large computational models called convolutional neural networks (CNNs) are time‐efficient and successful approaches for automated volumetric CT scan segmentation (Mortensen et al., 2019; Polymeri et al., 2020).

In this study, we aimed to design and test an AI‐based model to segment the common carotid arteries and examine whether it could segment faster than the manual approach and provide comparable data for tracer uptake, so that the AI‐based approach as support or replacement may serve to increase the routine clinical use of NaF‐PET for assessment of carotid atherosclerotic burden.

## 2 METHODS

### 2.1 Study design

CNNs were trained earlier to segment the carotids automatically. A single image analyser performed manual segmentation of the heart and aorta in 49 participants, primarily included as a part of the ‘Cardiovascular Molecular Calcification Assessed by 18F‐NaF PET/CT’ (CAMONA) study (Blomberg et al., 2014, 2015, 2017). The carotids were segmented in a way to encompass the artery wall and the inner blood pool. The accuracy of the automated segmentations was assessed by comparison with measurements obtained by manual segmentation in the same 49 subjects. We examined intra and interoperator variability with the manual approach by repeated manual segmentation in 25 randomly selected scans performed by the same operator and by two independent operators, respectively. The random subjects were selected using RandList software. The CNN‐based segmentation procedure has an inborn 100% repeatability (Trägårdh et al.).

### 2.2 Study population

The CAMONA study, conducted 2012–2014, included 89 healthy individuals with low cardiovascular disease risk recruited from the blood bank of Odense University Hospital or via local advertisement (Blomberg et al., 2017). Individuals considered healthy if they had no history of malignant diseases, immunodeficiency syndromes, autoimmune diseases, alcohol or substance abuse or cardiovascular diseases. They were preselected by age and gender to guarantee a balanced inclusion of both genders aged 20–29, 30–39, 40–49, 50–59, and 60 years or older. Also, 50 patients with suspected angina pectoris referred to the Department of Cardiology at Odense University Hospital for coronary angiography were included as the angina pectoris group. All original 89 + 50 subjects were invited to have a 2‐year follow‐up NaF‐PET/computed tomography (PET/CT) scan. However, despite direct inquiries, only 29 healthy controls and 20 patients responded. It is their basic NaF‐PET/CT scans, which constitute the material for the current assessment of the performance of CNN‐based segmentation.

### 2.3 Image analysis

For quantitative manual and automated analysis of the carotids, the Research Consortium for Medical Image Analysis (RECOMIA [https://www.recomia.org/]) was used. The carotids were segmented from the origin (brachiocephalic artery for right and arch of aorta for left carotid artery) to the end of the bifurcation. VOIs were formed by stacking manually defined region of interests (ROIs) covering the whole carotid arteries in the CT images of each participant to segment the carotids. The manual ROI determination contained the carotid arteries (artery wall and inner blood pool), excluding the vertebral bones and their uptake halo from the defined ROIs. Quantitative assessment was done by determining the segmented VOI volume (Vol) in ml and generating standardized uptake values (SUVs) for NaF uptake (in g/ml) in each VOI. SUVmean was the average SUV of all VOIs within VOI, SUVmax the highest SUV of all voxels in these VOIs, and SUVtotal the SUVmean multiplied in Vol of the VOI.

### 2.4 CNN segmentations

For the automatic segmentation, a fully convolutional CNN with the same structure as the 3D U‐Net (Çiçek et al., 2016) was trained. The 3D U‐Net is designed to have a large receptive field while still being able to use high‐resolution information. This is achieved by using max‐pooling downsampling, upconvolution upsampling and skip connections to process the input image on four different resolutions. The CNN takes a 100 × 100 × 100 Vol of voxels, where each voxel has a size of 3.0 × 1.37 × 1.37 mm, as input. For this input size, the CNN outputs an estimated class probability for each voxel of a 12 × 12 × 12 Vol at the centre of the input patch; in this case the classes are carotid left, carotid right and background.

For training, a set of 50 CT scans with annotations of the carotids from an external database was used. This data set was created during a previous project (Trägårdh et al., 2020) and the scans were separate from the 49 NaF‐PET/CT scans used for the main study. These 50 scans were then divided into a training set of 40 scans and a validation set of 10 scans. The loss function used was categorical cross‐entropy and the optimization was done using the Adam method (Kingma & Ba, 2014) with Nesterov momentum.

To produce the automatic carotid segmentations, the trained CNN was applied to the whole CT‐scan resulting in an initial segmentation. For postprocessing, the largest almost connected component was extracted. Almost refers to the fact that a distance of 20 mm between small segmentation components is allowed for them to still count as connected. To avoid having areas of high activity originating from surrounding bones which may strongly influence the SUV statistics, SUV leakage removal was performed. In detail, areas with SUV above a threshold (two standard deviations (SD) above the mean SUV in the carotids) and in which the closest activation maximum was located in the bones, were removed from the segmentation. The segmentation of the bones was done using an additional segmentation tool available on the RECOMIA platform (Trägårdh et al., 2020).

### 2.5 Statistical analysis

Frequency (percentage) and mean ± SD were used to express descriptive statistics. Bland‐Altman plots were used to assess the agreement between variables in pairwise segmentations (Carkeet, 2015; Gerke, 2020). The mean differences (bias) and the upper and lower Limits of Agreement (LoA) were calculated for the two methods. The Sørensen−Dice coefficient (SDC) was calculated to gauge the similarity of CNN and manual segmentations in the common carotids segmentation (Zijdenbos et al., 1994).

## 3 RESULTS

**Figure 1:**
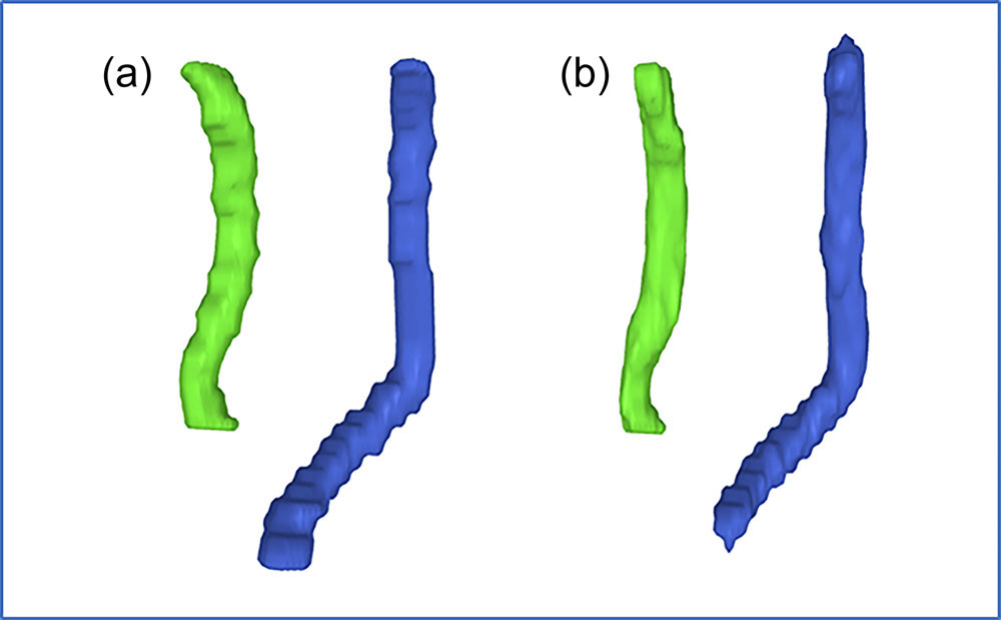
A three‐dimentional reconstruction of manual (a) and CNN‐based (b) common carotids segmentation in the same patient (right common carotid in light green and left common carotid in blue).

**Figure 2:**
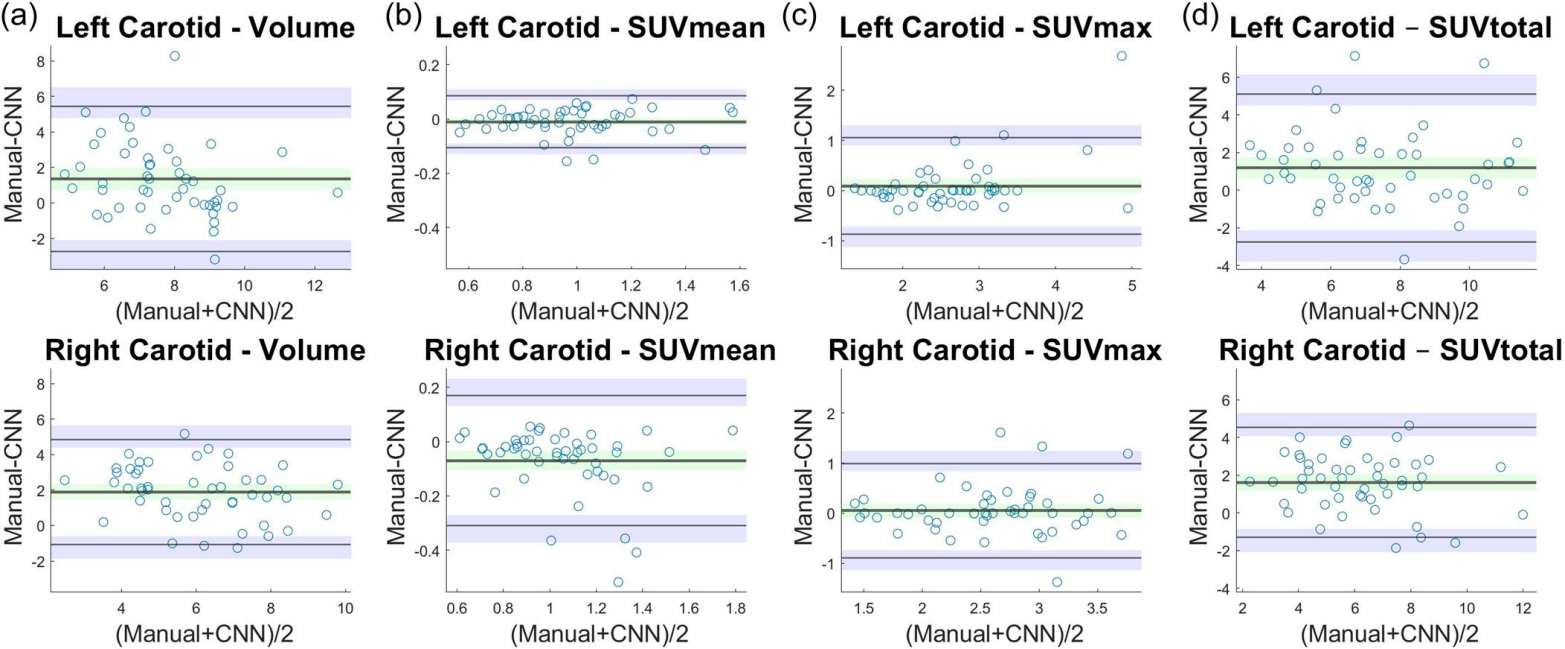
Bland−Altman plots for differences between Volume (a), SUVmean (b), SUVmax (c), and SUVtotal (d) obtained by Manual and CNN‐based segmentation (Manual–CNN) plotted against average ((Manual + CNN)/2) in left (upper panel) and right (lower panel) common carotids (*n* = 49). The estimated bias of one method relative to the other is the mean difference between values obtained by the two methods shown as a thick black horizontal line in the centre with its 95% confidence interval (green shade), whereas the Limits of Agreement are indicated by the thin black horizontal line lines with their 95% confidence interval (purple shades). CNN, convolutional neural network.

The mean age (±SD) of the subjects was 52 ± 12 years, ranging from 21 to 75. Twenty‐six (53%) were male. The mean height and weight of the subjects were 173.1 ± 9.1 cm and 82 ± 20.5 kg. An example of CNN versus manual segmentation is shown in Figure 1. The time required to manually segment the left and right common carotids was about 20 min compared to less than a minute with the CNN‐based method. The extracted data in manual and CNN‐based methods is presented in Table 1. Bland−Altman plots displaying differences between Vol, SUVmean, SUVmax, SUVtotal values obtained by manual and CNN‐based segmentation in the left and right common carotids are shown in Figure 2. Bland−Altman plots exhibited symmetrical difference distributions around the x‐axis for the paired differences, and variance homogeneity was observed over the measurement range. The outer confidence limits of Bland−Altman LoAs in left common carotid were for Vol −3.78 and 6.46 ml, for SUVmean −0.13 and 0.11 ml, for SUVmax −1.11 and 1.29 ml and for SUVtotal −3.74 and 6.1 ml. The outer confidence limits of the right common carotid LoAs were for Vol −1.83 and 5.6 ml, for SUVmean −0.37 and 0.23 ml, for SUVmax −1.12 and 1.23 ml and for SUVtotal −2.04 and 5.26 ml. The values for intraoperator, interoperator and manual‐CNN variability in the left and right carotid artery are shown in Table 2 and Table 3, respectively. The mean Vol deviation at repeat manual segmentation was 14% and 27%, respectively, in left and right common carotids. The mean SDC for left and right common carotids are shown in Table 4; the CNN versus manual SDC and Interobserver SDC were not statistically different in left (*p* = 0.66) and right (*p* = 0.59) common carotids.

**Table 1:** Quantities measured by manual and CNN‐based segmentation

Segment	Parameter	All subjects (*n* = 49)			*p* Value
		Manual	CNN	Difference (95% CI)	Manual versus CNN
Left carotid	Volume	8.36 ± 1.62	7.02 ± 2.11	1.34 (0.74−1.94)	<0.001
	SUVmean	0.97 ± 0.23	0.98 ± 0.23	−0.01 (−0.02 to 0.01)	0.19
	SUVmax	2.68 ± 0.91	2.59 ± 0.71	0.09 (−0.05 to 0.23)	0.14
	SUVtotal	8.02 ± 2.28	6.84 ± 2.47	1.18 (0.6−1.76)	<0.001
Right carotid	Volume	6.96 ± 1.65	5.08 ± 2.03	1.89 (1.45−2.32)	<0.001
	SUVmean	1.02 ± 0.23	1.09 ± 0.27	−0.07 (−0.1 to −0.03)	<0.001
	SUVmax	2.62 ± 0.67	2.57 ± 0.64	0.05 (−0.09 to 0.19)	0.45
	SUVtotal	7 ± 2.03	5.39 ± 2.03	1.61 (1.18−2.04)	<0.001

**Table 2:** Differences between two left common carotid segmentations: Intraoperator, Interoperator and manual–CNN variability

Parameter	Intraoperator (*n* = 25)		Interoperator (*n* = 25)		Manual–CNN (*n* = 49)	
	Mean ± SD	LoA	Mean ± SD	LoA	Mean±SD	LoA
Volume	−0.69 ± 0.98	−2.13 to 1.06	1.73 ± 1.59	−1.39 to 4.85	1.33 ± 2.06	−2.71 to 5.39
SUVmean	0 ± 0.02	−0.04 to 0.04	−0.01 ± 0.06	−0.13 to 0.11	−0.01 ± 0.05	−0.08 to 0.1
SUVmax	0 ± 0.3	−0.58 to 0.59	0.02 ± 0.31	−0.58 to 0.63	0.09 ± 0.48	−0.86 to 1.04
SUVtotal	−0.7 ± 0.9	−3.15 to 0.53	1.44 ± 1.59	−1.67 to 4.56	1.18 ± 1.99	−2.71 to 5.07

Abbreviations: LoA, Limits of Agreement, SD, standard deviation.

**Table 3:** Differences between two right common carotid segmentations: Intraoperator, Interoperator and manual‐CNN variability

Parameter	Intraoperator (*n* = 25)		Interoperator (*n* = 25)		Manual‐CNN (*n* = 49)	
	Mean ± SD	LoA	Mean ± SD	LoA	Mean ± SD	LoA
Volume	−0.94 ± 1.13	−3.15 to 1.27	1.28 ± 1.91	−2.46 to 5.02	1.89 ± 1.5	−1.05 to 4.82
SUVmean	−0.01 ± 0.07	−0.15 to 0.11	0.01 ± 0.06	−0.12 to 0.13	−0.07 ± 0.12	−0.31 to 0.17
SUVmax	−0.03 ± 0.34	−0.69 to 0.62	−0.01 ± 0.34	−0.68 to 0.66	0.05 ± 0.47	−0.88 to 0.98
SUVtotal	−1.06 ± 1.23	−3.47 to 1.34	1.09 ± 1.9	−2.64 to 4.82	1.61 ± 1.47	−1.28 to 4.5

Abbreviations: LoA, Limits of Agreement; SD, standard deviation.

**Table 4:** The mean SDC ( ± SD) for left and right common carotids

	Intraobserver	Interobserver	CNN versus manual
Left	0.84 ± 0.05	0.73 ± 0.08	0.72 ± 0.1
Right	0.77 ± 0.09	0.65 ± 0.13	0.63 ± 0.16

Abbreviations: CNN, convolutional neural network; SDC, Sørensen−Dice coefficient; SD, standard deviation.

## 4 DISCUSSION

### 4.1 Principal findings

The CNN‐derived measures in the left and right common carotids were 0%−15% and 0%−24% lower than the manually derived values, mainly reflecting deviation in the Vol, whereas values for SUVmean obtained with the two approaches were very similar in both left and right common carotids. Moreover, CNN‐based segmentation and quantification was much faster, observer‐independent and had a maximal deviation of 13% and 16% at repeat manual segmentation of the left and right common carotids, respectively.

### 4.2 Strengths and weaknesses of the study

An important practical limitation of quantitative PET studies is the manual or semiautomatic segmentation of the scans. These are time‐consuming processes requiring an experienced image analyser to define the VOIs in the scans, which alone may sometimes last half an hour or more when it comes to carotid artery segmentation. This is a major issue in many clinical and research studies and one of the reasons why we decided to turn to AI and deep learning, computer‐based tools that have improved several aspects of diagnosis in the medical field. Thus, nuclear cardiology has used AI to facilitate attenuation correction (Irkle et al., 2015) or automate myocardial perfusion reports (Kashiwazaki et al., 2018). We developed and tested CNN‐based models for segmentation of the common carotids in NaF‐PET/CT studies. To our knowledge, they are the first reported models used for this purpose. Our model was able to segment much faster than what is possible with the manual approach even after training based on a very small number of scans, similar to what we have previously shown with regard to CNN‐based segmentation of the aorta and the heart (Piri, Edenbrandt, Larsson, Enqvist, Nøddeskou‐Fink et al., 2021; Piri, Edenbrandt, Larsson, Enqvist, Skovrup et al. 2021).

The most important strength of this CNN‐based model was the ability to segment common carotids comparable to the manual segmentation, which is difficult even for trained image analysers. Noncontrast CT used in hybrid imaging modalities, such as NaF‐PET/CT, is not optimal for studying cardiovascular structures since distinguishing different anatomic structures is difficult in the absence of intravenous contrast. Second, common carotids are anatomic structures prone to relatively large interindividual variation. Therefore, the ability of this CNN‐based model to distinguish common carotids beside other similar structures such as jugular vein, lymphatic nodes and muscles was quite satisfactory.

The main limitation of the CNN‐based segmentation was some inaccuracy of the segmented VOIs due to variation in the vascular system, especially the right common carotid, the origin of which from the brachiocephalic artery is rather difficult to identify. Manual segmentation of such variations could be challenging as well (Ntaios et al., 2021). The CNN‐based model is much faster, but we cannot document that it is also more accurate than the manual one, since there is no infallible reference to compare with. We can only point to its superior reproducibility, observer‐independence and apparently also relative independence of PET/CT scanner type and make (Boellaard et al., 2015; Boellaard et al., 2019; Hagiwara et al., 2020). We expect that it is a matter of time before CNN‐based segmentation will outperform the manual segmentation, for the simple reason that it will continuously learn and improve as more and more scans of patients with diverse disorders and variable anatomical structures have been examined for training purposes. Finally, the proximity of high uptake structures, such as the sternum or vertebral bones, is another challenging factor, which we tried to correct for in different ways with the two methods. Also at this point, it is expected that the CNN‐based methodology will take the lead based on multiple upcoming training examples and a never‐ending apprenticeship.

### 4.3 Possible mechanisms and implications

The most probable explanation for the difference between CNN‐based and manual segmentation is the similarity of density between anatomic structures near to the common carotids. This similarity may lead to mislocating the common carotid arteries, either by inclusion of adjacent structures in the VOIs or exclusion of common carotid tissue. So, the Vol of segmented VOIs was in general somewhat higher with the manual method. This would also lead to a different SUVtotal, but not influence SUVmean, which remained robust to minor over‐ or under‐segmentation and, thus, in our view stand out as the relevant measure of the atherosclerotic burden also in the carotids as argued elsewhere with regard to the aorta and entire heart (Høilund‐Carlsen et al., 2019; Piri, Edenbrandt, Larsson, Enqvist, Nøddeskou‐Fink et al., 2021; Piri, Edenbrandt, Larsson, Enqvist, Skovrup et al. 2021). However, from here and to recommend NaF‐PET/CT in all stroke/TIA patients or all patients examined in this way, we have to await the outcome of an extended clinical application, which is precisely what CNN‐based quantification makes possible.

### 4.4 Unanswered questions and future research

The present work was mainly a feasibility study elucidating if the CNN approach can segment the carotids from the non‐contrast CT part of an ordinary PET/CT scan and yield SUVmean measures of NaF uptake comparable to those obtained manually. That this can be done in 1 min is a huge progress which opens for routine application. However, to what degree it will impact clinically, only time and prospective interventional and longitudinal studies can show. It depends largely on whether arterial NaF uptake is a precursor of macrocalcification that is detectable by ultrasound and CT, as certain animal and human studies indicate (Høilund‐Carlsen et al., 2020; McKenney‐Drake et al., 2018). If so, it is foreseeable that the method will be applied in patients with suspected stroke/TIA, probably looking for NaF uptake not only in the carotids but in the entire preceding part of the arterial system.

It is unknown how well the AI‐based approach will work in patients with major anatomical variations and to what degree it can result in reliable estimates of changes over time or due to intervention. The CNN‐based model presented here is preliminary and probably the first to demonstrate feasibility of AI‐based common carotid segmentation. The presented results were acquired after training on a very limited amount of learning material, a circumstance which gives reason to believe that the AI‐based approach will after further training in more extreme cardiovascular cases gradually become the mainstay of image analysis in patients with suspected or known atherosclerotic disease.

## 5 CONCLUSION

The new CNN‐based model for automated segmentation of common carotids was fast and reproduced values for common carotid NaF uptake that were comparable to those acquired by manual segmentation. With increased ongoing learning we expect that the CNN‐based processing of NaF‐PET/CT scans will be a valuable time‐saving addition to routine assessment of the atherosclerotic burden in the common carotids and other major arteries.

## CONFLICT OF INTEREST

The authors declare no conflict of interest.

## Data Availability

Data is available upon reasonable request from the corresponding author.

## References

[cpf12793-bib-0001] Barquera, S. , Pedroza‐Tobías, A. , Medina, C. , Hernández‐Barrera, L. , Bibbins‐Domingo, K. , Lozano, R. et al. (2015) Global overview of the epidemiology of atherosclerotic cardiovascular disease. Archives of Medical Research, 46, 328–338. 26135634 10.1016/j.arcmed.2015.06.006

[cpf12793-bib-0002] Blomberg, B.A. , de Jong, P.A. , Thomassen, A. , Lam, M.G.E. , Vach, W. , Olsen, M.H. et al. (2017) Thoracic aorta calcification but not inflammation is associated with increased cardiovascular disease risk: results of the CAMONA study. European Journal of Nuclear Medicine and Molecular Imaging, 44, 249–258. 27796543 10.1007/s00259-016-3552-9 PMC5214929

[cpf12793-bib-0003] Blomberg, B.A. , Thomassen, A. , de Jong, P.A. , Simonsen, J.A. , Lam, M.G.E.H. , Nielsen, A.L. et al. (2015) Impact of personal characteristics and technical factors on quantification of sodium 18F‐fluoride uptake in human arteries: prospective evaluation of healthy subjects. Journal of Nuclear Medicine, 56, 1534–1540. 26205304 10.2967/jnumed.115.159798

[cpf12793-bib-0004] Blomberg, B.A. , Thomassen, A. , Takx, R.A.P. , Hildebrandt, M.G. , Simonsen, J.A. , Buch‐Olsen, K.M. et al. (2014) Delayed 18 F‐fluorodeoxyglucose PET/CT imaging improves quantitation of atherosclerotic plaque inflammation: results from the CAMONA study. Journal of Nuclear Cardiology, 21, 588–597. 24633502 10.1007/s12350-014-9884-6

[cpf12793-bib-0005] Boellaard, R. , Delgado‐Bolton, R. , Oyen, W.J.G. , Giammarile, F. , Tatsch, K. & Eschner, W. et al. (2015) FDG PET/CT: EANM procedure guidelines for tumour imaging: version 2.0. European Journal of Nuclear Medicine and Molecular Imaging, 42, 328–354. 25452219 10.1007/s00259-014-2961-x PMC4315529

[cpf12793-bib-0006] Boellaard, R. , Sera, T. , Kaalep, A. , Hoekstra, O.S. , Barrington, S.F. & Zijlstra, J.M. (2019) Updating PET/CT performance standards and PET/CT interpretation criteria should go hand in hand. EJNMMI Research, 9, 95. 31664529 10.1186/s13550-019-0565-y PMC6820631

[cpf12793-bib-0007] Carkeet, A. (2015) Exact parametric confidence intervals for Bland‐Altman limits of agreement. Optometry and Vision Science, 92, e71–e80. 25650900 10.1097/OPX.0000000000000513

[cpf12793-bib-0008] Chambless, L.E. , Folsom, A.R. , Clegg, L.X. , Sharrett, A.R. , Shahar, E. , Nieto, F.J. et al. (2000) Carotid wall thickness is predictive of incident clinical stroke: the atherosclerosis risk in communities (ARIC) study. American Journal of Epidemiology, 151, 478–487. 10707916 10.1093/oxfordjournals.aje.a010233

[cpf12793-bib-0009] Çiçek, Ö. , Abdulkadir, A. , Lienkamp, S.S. , Brox, T. & Ronneberger, O. (2016) 3D U‐Net: learning dense volumetric segmentation from sparse annotation, International CConference on Medical Image Computing and Computer‐assisted Intervention. Springer, pp. 424–432.

[cpf12793-bib-0010] Derlin, T. , Wisotzki, C. , Richter, U. , Apostolova, I. , Bannas, P. & Weber, C. et al. (2011) In vivo imaging of mineral deposition in carotid plaque using 18F‐sodium fluoride PET/CT: correlation with atherogenic risk factors. Journal Nuclear Medicine, 52, 362–368. 10.2967/jnumed.110.081208 21321276

[cpf12793-bib-0011] Dou, Q. , Yu, L. , Chen, H. , Jin, Y. , Yang, X. , Qin, J. et al. (2017) 3D deeply supervised network for automated segmentation of volumetric medical images. Medical Image Analysis, 41, 40–54. 28526212 10.1016/j.media.2017.05.001

[cpf12793-bib-0012] Enos, W.F. (1955) Pathogenesis of coronary disease in American soldiers killed in Korea. Journal of the American Medical Association, 158, 912–914. 14381267 10.1001/jama.1955.02960110018005

[cpf12793-bib-0013] Gerke, O. (2020) Reporting standards for a Bland–Altman agreement analysis: A review of methodological reviews. Diagnostics, 10, 334. 32456091 10.3390/diagnostics10050334 PMC7278016

[cpf12793-bib-0014] Hagiwara, A. , Fujita, S. , Ohno, Y. & Aoki, S. (2020) Variability and standardization of quantitative imaging: monoparametric to multiparametric quantification, radiomics, and artificial intelligence. Investigative Radiology, 55, 601–616. 32209816 10.1097/RLI.0000000000000666 PMC7413678

[cpf12793-bib-0015] Holman, R.L. , McGILL HC, Jr. , Strong, J.P. & Geer, J.C. (1958) The natural history of atherosclerosis: the early aortic lesions as seen in new orleans in the middle of the of the 20th century. The American Journal of Pathology, 34, 209–235. 13520905 PMC1934740

[cpf12793-bib-0016] Høilund‐Carlsen, P.F. , Edenbrandt, L. & Alavi, A. (2019) Global disease score (GDS) is the name of the game! European Journal of Nuclear Medicine and Molecular Imaging, 46, 1768–1772. 31183636 10.1007/s00259-019-04383-8 PMC6647113

[cpf12793-bib-0017] Høilund‐Carlsen, P.F. , Piri, R. , Constantinescu, C. , Iversen, K.K. , Werner, T.J. , Sturek, M. et al. (2020) Atherosclerosis imaging with 18F‐Sodium fluoride PET. Diagnostics, 10, 852. 33092250 10.3390/diagnostics10100852 PMC7590213

[cpf12793-bib-0018] Høilund‐Carlsen, P.F. , Piri, R. , Gerke, O. , Edenbrandt, L. & Alavi, A. (2021) Assessment of Total‐Body atherosclerosis by PET/Computed tomography. PET Clinics, 16, 119–128. 33160930 10.1016/j.cpet.2020.09.013

[cpf12793-bib-0019] Irkle, A. , Vesey, A.T. , Lewis, D.Y. , Skepper, J.N. , Bird, J.L.E. , Dweck, M.R. et al. (2015) Identifying active vascular microcalcification by 18 f‐sodium fluoride positron emission tomography. Nature Communications, 6, 7495. 10.1038/ncomms8495 PMC4506997 26151378

[cpf12793-bib-0020] Kashiwazaki, D. , Yamamoto, S. , Akioka, N. , Kuwayama, N. , Noguchi, K. & Kuroda, S. (2018) Inflammation coupling between unstable carotid plaque and Spleen—A 18F‐Fluorodeoxyglucos positron emission tomography study. Journal of Stroke and Cerebrovascular Diseases, 27, 3212–3217. 30087079 10.1016/j.jstrokecerebrovasdis.2018.07.020

[cpf12793-bib-0021] Kingma, D.P. & Ba, J. (2014) Adam: a method for stochastic optimization. arXiv preprint arXiv:14126980.

[cpf12793-bib-0022] Lindgren Belal, S. , Sadik, M. , Kaboteh, R. , Enqvist, O. , Ulén, J. , Poulsen, M.H. et al. (2019) Deep learning for segmentation of 49 selected bones in CT scans: first step in automated PET/CT‐based 3D quantification of skeletal metastases. European Journal of Radiology, 113, 89–95. 30927965 10.1016/j.ejrad.2019.01.028

[cpf12793-bib-0023] Lorenz, M.W. , von Kegler, S. , Steinmetz, H. , Markus, H.S. & Sitzer, M. (2006) Carotid intima‐media thickening indicates a higher vascular risk across a wide age range: prospective data from the carotid atherosclerosis progression study (CAPS). Stroke, 37, 87–92. 16339465 10.1161/01.STR.0000196964.24024.ea

[cpf12793-bib-0024] McGill HC, Jr. (1968) Fatty streaks in the coronary arteries and aorta. Laboratory Investigation; A Journal of Technical Methods and Pathology, 18, 560–564. 5681198

[cpf12793-bib-0025] McKenney‐Drake, M.L. , Moghbel, M.C. , Paydary, K. , Alloosh, M. , Houshmand, S. , Moe, S. et al. (2018) 18 F‐NaF and 18 F‐FDG as molecular probes in the evaluation of atherosclerosis. European Journal of Nuclear Medicine and Molecular Imaging, 45, 2190–2200. 29978245 10.1007/s00259-018-4078-0 PMC6182398

[cpf12793-bib-0026] Mortensen, M.A. , Borrelli, P. , Poulsen, M.H. , Gerke, O. , Enqvist, O. , Ulén, J. et al. (2019) Artificial intelligence‐based versus manual assessment of prostate cancer in the prostate gland: a method comparison study. Clinical Physiology and Functional Imaging, 39, 399–406. 31436365 10.1111/cpf.12592

[cpf12793-bib-0027] Ntaios, G. , Sagris, D. , Strambo, D. , Perlepe, K. , Sirimarco, G. , Georgiopoulos, G. et al. (2021) Carotid atherosclerosis and patent foramen ovale in embolic stroke of undetermined source. Journal of Stroke and Cerebrovascular Diseases, 30, 105409. 33137616 10.1016/j.jstrokecerebrovasdis.2020.105409

[cpf12793-bib-0028] World Health Organization . (2019) World health statistics 2019: monitoring health for the SDGs, sustainable development goals. World Health Organization.

[cpf12793-bib-0029] Piri, R. , Edenbrandt, L. , Larsson, M. , Enqvist, O. , Nøddeskou‐Fink, A.H. , Gerke, O. et al. (2021) Aortic wall segmentation in ^18^F‐sodium fluoride PET/CT scans: head‐to‐head comparison of artificial intelligence‐based versus manual segmentation. Journal of Nuclear Cardiology, 29, 2001–2010. 33982202 10.1007/s12350-021-02649-z

[cpf12793-bib-0030] Piri, R. , Edenbrandt, L. , Larsson, M. , Enqvist, O. , Skovrup, S. , Iversen, K.K. et al. (2021) “Global” cardiac atherosclerotic burden assessed by artificial intelligence‐based versus manual segmentation in ^18^F‐sodium fluoride PET/CT scans: head‐to‐head comparison. Journal of Nuclear Cardiology, 29, 2531–2539. 34386861 10.1007/s12350-021-02758-9

[cpf12793-bib-0031] Polymeri, E. , Sadik, M. , Kaboteh, R. , Borrelli, P. , Enqvist, O. , Ulén, J. et al. (2020) Deep learning‐based quantification of PET/CT prostate gland uptake: association with overall survival. Clinical Physiology and Functional Imaging, 40, 106–113. 31794112 10.1111/cpf.12611 PMC7027436

[cpf12793-bib-0032] Prabhakaran, S. , Singh, R. , Zhou, X. , Ramas, R. , Sacco, R.L. & Rundek, T. (2007) Presence of calcified carotid plaque predicts vascular events: the Northern manhattan study. Atherosclerosis, 195, e197–e201. 17482197 10.1016/j.atherosclerosis.2007.03.044 PMC6286814

[cpf12793-bib-0033] Raynor, W. , Houshmand, S. , Gholami, S. , Emamzadehfard, S. , Rajapakse, C.S. , Blomberg, B.A. et al. (2016) Evolving role of molecular imaging with 18 F‐Sodium fluoride PET as a biomarker for calcium metabolism. Current osteoporosis reports, 14, 115–125. 27301549 10.1007/s11914-016-0312-5

[cpf12793-bib-0034] Sorci, O. , Batzdorf, A.S. , Mayer, M. , Rhodes, S. , Peng, M. , Jankelovits, A.R. et al. (2020) 18F‐sodium fluoride PET/CT provides prognostic clarity compared to calcium and Framingham risk scoring when addressing whole‐heart arterial calcification. European Journal of Nuclear Medicine and Molecular Imaging, 47, 1678–1687. 31734781 10.1007/s00259-019-04590-3

[cpf12793-bib-0035] Trägårdh, E. , Borrelli, P. , Kaboteh, R. , Gillberg, T. , Ulén, J. , Enqvist, O. et al. (2020) RECOMIA—a cloud‐based platform for artificial intelligence research in nuclear Medicine and radiology. EJNMMI Physics, 7, 51. 32754893 10.1186/s40658-020-00316-9 PMC7403290

[cpf12793-bib-0036] Wu, X.H. , Chen, X.Y. , Fan, Y.H. , Leung, T.W.H. & Wong, K.S. (2017) High extent of intracranial carotid artery calcification is associated with downstream microemboli in stroke patients. Journal of Stroke and Cerebrovascular Diseases, 26, 442–447. 27818028 10.1016/j.jstrokecerebrovasdis.2016.10.007

[cpf12793-bib-0037] Zijdenbos, A.P. , Dawant, B.M. , Margolin, R.A. & Palmer, A.C. (1994) Morphometric analysis of White matter lesions in MR images: method and validation. IEEE Transactions on Medical Imaging, 13, 716–724. 18218550 10.1109/42.363096

